# Selective Serotonin Reuptake Inhibitors: A Review of its Effects on Intraocular Pressure

**DOI:** 10.2174/157015908787386104

**Published:** 2008-12

**Authors:** Ciro Costagliola, Francesco Parmeggiani, Francesco Semeraro, Adolfo Sebastiani

**Affiliations:** 1 Dipartimento di Scienze per la Salute, Università degli Studi del Molise, Campobasso, Italy; 2 Dipartimento di Discipline Medico-Chirurgiche della Comunicazione e del Comportamento, Sezione di Oculistica, Università degli Studi di Ferrara, Ferrara, Italy; 3 Clinica Oculistica, Università degli Studi di Brescia, Brescia, Italy

**Keywords:** Fluoxetine, sertraline, paroxetine, fluvoxamine, citalopram, escitalopram, intraocular pressure, side effects.

## Abstract

The increase in serotonin (5-HT) neurotransmission is considered to be one of the most efficacious medical approach to depression and its related disorders. The selective serotonin reuptake inhibitors (SSRIs) represent the most widely antidepressive drugs utilized in the medical treatment of depressed patients. Currently available SSRIs include fluoxetine, sertraline, paroxetine, fluvoxamine, citalopram and escitalopram. The primary SSRIs pharmacological action’s mechanism consists in the presynaptic inhibition on the serotonin reuptake, with an increased availability of this amine into the synaptic cleft. Serotonin produces its effects as a consequence of interactions with appropriate receptors. Seven distinct families of 5-HT receptors have been identified (5-HT_1_ to 5-HT_7_), and subpopulations have been described for several of these. The interaction between serotonin and post-synaptic receptors mediates a wide range of functions. The SSRIs have a very favorable safety profile, although clinical signs of several unexpected pathologic events are often misdiagnosed, in particular, those regarding the eye. In all cases reported in the literature the angle-closure glaucoma represents the most important SSRIs-related ocular adverse event. Thus, it is not quite hazardous to hypothesize that also the other reported and unspecified visual disturbances could be attributed - at least in some cases - to IOP modifications. The knowledge of SSRIs individual tolerability, angle-closure predisposition and critical IOP could be important goals able to avoid further and more dangerous ocular side effects.

## 1. SEROTONIN AND 5-HT RECEPTORS

**Fig. (1):**
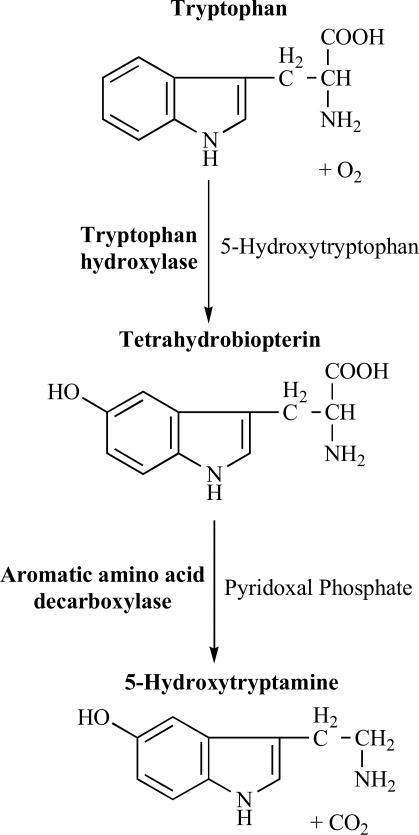
The biosynthesis of serotonin from the amino acid tryptophan.

**Fig. (2):**
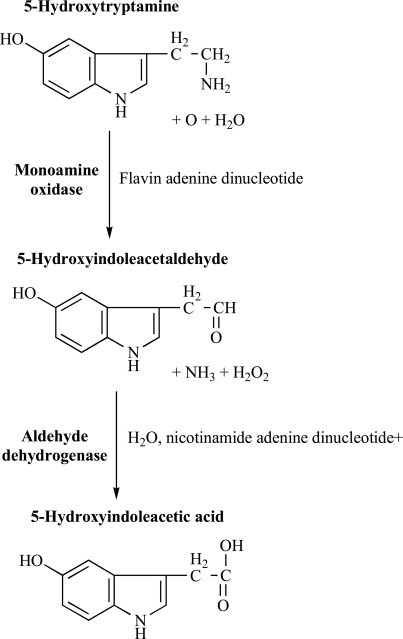
The catabolism of serotonin.

Serotonin (5-hydroxytryptamine, 5-HT) is widely distributed in animals and plants, occurring in vertebrates, fruits, nuts, and venoms. In 1948, Rapport and co-workers were the first to isolate this vasoconstrictor, released from platelets in clotting blood, which subsequently named serotonin [119]. 5-HT is a biogenic monoamine that produces its effects through a variety of membrane-bound receptors. Although serotonin may be obtained from a variety of dietary sources, in humans endogenous 5-HT is synthesized *in situ* from tryptophan, its amino acid precursor, through two steps. First, tryptophan is hydroxylated to 5-hydroxytryptophan by tryptophan hydroxylase; then, 5-hydroxytryptophan is decarboxylated to 5-hydroxytryptamine (Fig. **1**). The hydroxylation of tryptophan is the rate-limiting step in this process. Tryptophan hydroxylase is only half saturated with its substrate, which suggests that increasing the level of tryptophan should increase the 5-HT synthesis [30, 124]. Both dietary and endogenous 5-HT are rapidly metabolized and inactivated by monoamine oxidase (MAO) and aldehyde dehydrogenase to the major metabolite, 5-hydroxy-indoleacetic acid (5-HIAA), even if most of the 5-HT released into the post-synaptic space is removed by the neuron through a re-uptake mechanism (Fig. **2**). There are two important types of MAO, which possess different preferential affinity to serotonin (MAO-A) or dopamine (MAO-B) [124]. 5-HT is not easily transported across the blood-brain barrier; conversely, tryptophan is actively transported across the blood-brain barrier from circulation [25].

Despite its relatively simple chemical structure, serotonin exhibits very complex properties. Based on the similarity with noradrenaline (NA) and dopamine, it is not surprising that 5-HT, like its catecholamine counterparts, possesses a broad spectrum of different psychological effects. The actions of 5-HT are realized by three major mechanisms: *i*) diffusion; *ii*) metabolism; and *iii*) uptake back into the synaptic cleft, through the action of specific amine membrane transported systems. Thus, the actions of 5-HT can be theoretically modulated by agents that play a role at different steps: (I) stimulate or inhibit its biosynthesis; (II) block its storage; (III) stimulate or inhibit its release; (IV) mimic or inhibit its actions at its various post-synaptic receptors; (V) inhibit its uptake back into the nerve terminal; (VI) affect its metabolism [51, 124].

Serotonin has been found both in several non-neuronal districts of the human body (i.e. blood, cardiovascular system, gastrointestinal tract and eye), and in the central and peripheral nervous systems (CNS/PNS), where it works as neurotransmitter or hormone. 5-HT is involved in the regulation of numerous physiological processes. Serotonergic neurons, at the level of the CNS, are found in the raphe nuclei of the brainstem/pons region; from here, 5-HT is able to influence most areas of the CNS, modulating a wide variety of sensory, motor, and cortical functions [101]. 5-HT has been shown to reset or phase-shift the circadian rhythm, controlling the endogenous clock located at the levels of the suprachiasmatic nuclei of the hypothalamus. In the PNS, serotonin is involved in such different functions as the initiation of activity in primary afferent nociceptors; 5-HT is an effector on various types of smooth muscle, functioning also at the level of ciliary muscle and sphincter of the pupil. This monoamine is implicated in the regulation of enteric reflexes, increasing the gastrointestinal motility, in the modulation of vascular smooth muscle contraction, constricting the large vessels and/or producing both contraction and relaxation of coronary artery in mammals. Moreover, 5-HT represents a precursor for melatonin production in the pineal gland and in the lens; it is an agent that contributes to the modulation of platelet shape change, enhancing the platelet aggregation (homeostasis), and concurs to the regulation of lymphocyte cyto-toxicity and phagocytosis [2, 51, 66, 108, 112, 124].

Adequate serotonin levels regulate sleep, mood, food intake and pain tolerance; whereas significant systemic or localized 5-HT alterations are thought to produce insomnia, increased food cravings, increased sensitivity to pain, poor body-temperature regulation, depressive and aggressive behaviors. Thus, 5-HT has been implicated in the etiology of miscellaneous physiopathologic conditions and diseases, i.e. depression, anxiety, social phobia, schizophrenia, obsessive-compulsive and panic disorders, migraine, intraocular pressure (IOP) modification, systemic hypertension, pulmonary hypertension, eating disorders, vomiting, and irritable bowel syndrome [28, 51, 66, 73, 108, 124, 132, 137, 138].

The chemical neurotransmitter serotonin produces its effects as a consequence of interactions with appropriate receptors. 5-HT is synthesized in neurons and stored in vesicles. Upon a nerve impulse, it is released into the synaptic cleft, where it interacts with multiple post-synaptic receptors, mediating a wide range of physiological functions. Seven distinct families of 5-HT receptors have been identified (5-HT_1_ to 5-HT_7_), and subpopulations have been described for several of these (Table **1**). The 5-HT receptor subtypes are expressed in distinct but often overlapping patterns and are coupled to different transmembrane-signaling mechanisms. Four 5-HT receptor families with defined functions, 5-HT_1_ to 5-HT_4_, are currently recognized. With the exception of the 5-HT_3_ receptor, the other subtypes of these receptors belong to the G-protein-coupled receptor (GPCR) superfamily, and are related to a predicted membrane topology which is composed of an extracellular N-terminal segment, linked to an intracellular C terminus by seven transmembrane-spanning segments. Otherwise, the 5-HT_3_ receptor is a ligand-gated ion channel that gates Na^+^ and K^+^ and has a predicted membrane topology similar to that of the nicotine cholinergic receptor. In the last two decades, most of the currently known 5-HT receptor populations have been identified and a high number of papers in the serotonin area has been published. These investigations have reported the cloning of several receptor populations previously known but not yet cloned, the development of novel agonists and antagonists with greater receptor subtypes selectivity, additional molecular biology studies (i.e. site-directed mutagenesis), and new pharmacologic and clinical findings. Evidence continues to emerge emphasizing the role of 5-HT receptors in various neuropsychiatric disorders. Anxiety, depression, schizophrenia, migraine, and drug abuse are at the top of this list. 5-HT receptors may also be involved in appetite control, aggression, sexual behavior, and cardiovascular disorders. Recently, working with always more specific methodological approach it has been documented that some medications, previously considered selective, are not as selective as originally hypothesized. In this way, it has been possible to understand both the therapeutic actions and some paradox effects related to the administration of these substances, as well as the occurrence of, otherwise unexplained, side effects. The multiple 5-HT receptor subtypes cloned to date represent one of the most complex family of known neurotransmitter receptors with, at least, fourteen distinct members [51, 66, 124].

## 2. ROLE OF SEROTONIN IN THE EYE

**Fig. (3):**
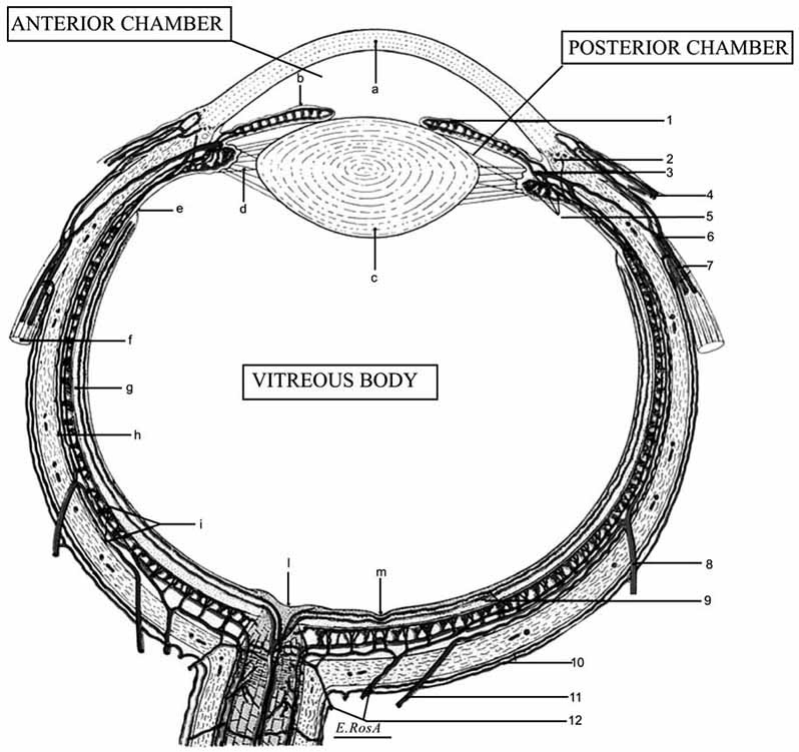
Schematic horizontal section of the human eye: anatomic structures and vasculature. **Legend:** a, cornea; b, iris; c, lens; d; suspensory ligament of the lens (ciliary zonule of Zinn); e, ora serrata; f, medial rectus muscle of the eye; g, retina; h, sclera; i, choroid; l, optic nerve head; m, central fovea of the macula; n, lamina cribrosa; o, irido-corneal angle. 1, minor circle of iris; 2, Schlemm's channel; 3, major circle of iris; 4, iuxtalimbic conjunctival vessels; ciliary circle; 6, anterior ciliary artery; 7, anterior ciliary arteries and veins; 8, vorticose vein; 9, retinal microvasculature; 10, episcleral arteries and veins; 11, long posterior ciliary artery; 12, short posterior ciliary arteries; 13, central retinal artery and vein; 14, perioptic nerve arteriolar anastomoses (circle of Haller and Zinn); 15, pial microarterioles.

In the mammalian eye (Fig. **3**), the distribution and the functions of serotonin have been mainly investigated by Osborne and his group at the Nuffield Laboratory of Ophthalmology in Oxford. Their researches represent a milestone to understand the physiopathology of this biogenic monoamine and have been the substantial core to build this review article. Serotonin has been found in different ocular structures depending on both CNS and PNS. Since 1948 when serotonin was first isolated, identified and synthesized, there has been an exponential growth in the information available on its biochemical, physiologic, and behavioral effects. Historically, initial researches were focused to define the pathways for synthesis and degradation of 5-HT and to develop of drugs interacting with these processes. In more recent times, the discovery and widespread clinical use of selective antidepressive medications, the pre-clinical delineation of the multiple 5-HT receptor subtypes together with their coupling to intracellular messenger systems, and the development of compounds selectively acting on these systems, have catalyzed an explosion of new information in this field. It is now clear that the 5-HT system is involved in a multitude of physiologic and behavioral processes. Shortly after the discovery of 5-HT as a potent vasoconstrictor agent in blood serum [119], this monoamine was found in many other tissues, including several districts of the mammalian eye [45, 102, 103, 108, 109, 129, 137, 138]. This led to the suggestion that serotonin could function as a neurotransmitter and/or a hormone in various central and peripheral mammalian tissues. The mammalian eye contains both central and peripheral neuronal tissues. The retina, as an out-growth of the brain, is considered as a part of the CNS. The other parts of the eye, namely the cornea, sclera, iris, ciliary muscle and processes, retinal pigment epithelium and choroid, receiving nerves that are derived from the PNS, as more closely pertinent to them [108]. The discovery of receptors for serotonin within the eye strongly suggests that 5-HT plays a functional role in the various ocular tissues (Table **2**). The diverse action of 5-HT are mediated by a variety of specific receptors and, as pointed out in the specific section, seven class of these receptors have been recognized on the basis of structural, transductional and functional characteristics [28, 108]. Detection of 5HT receptor subtype mRNAs in numerous human ocular tissues has been successfully performed by Sharif and Senchyna [129]. While it is difficult to associate these observations with functional evidence for the existence of the various 5HT receptors in these tissues in every case, these data provide a foundation for future research to discover the physiological and pharmacological relevance of the 5HT receptors in the human ocular tissues.

### 2.1. Cornea

The presence of functional serotonergic receptors in the rabbit cornea was firstly postulated by Neufeld and co-workers [98]. These authors have demonstrated that serotonin increases the level of cAMP in incubated rabbit corneas and that the corneal epithelium contains specific serotonergic receptors. These specific serotonergic receptors are present in the corneal epithelium and their activation by serotonin released from serotonergic neurons increases both the level of cAMP at the level of stromal cells and the chloride transport at the level of corneal epithelium [73]. The serotonergic receptors must be at a location posterior to that of the β-adrenergic receptors, which are on the anterior-surface of the apical cells. The serotonin response is partially inhibited by the β-adrenergic antagonist, timolol [105]. The investigations conducted in the experimental models strongly suggest that in the cornea exist at least two classes of serotonergic receptors: the first responsible for increased levels of cAMP, located at the level of stromal cells; whereas the second responds to serotonin by increasing chloride transport in the epithelial cells, without a concomitant elevation of cAMP levels [73, 96, 105, 109]. Chidlow and co-workers did not found evidence for the existence of 5-HT_1A_ and 5-HT_7_ receptors in the cornea which, on the contrary, are widely distributed in other ocular tissues [28]. It can be hypothesized that 5-HT_4_ and 5-HT_6_ subtypes are present in the corneal stroma; in fact, the effect of 5-HT in the stroma is antagonized by methysergide [105], a ligand known to be inactive on 5-HT_4_ receptors [99], and only weakly effective on 5-HT_6_ receptors [42]. Experimental evidences have demonstrated the implication of 5-HT_2_, 5-HT_3_ and 5-HT_4_ serotonin receptor subtypes in the chloride transport in various tissues [28, 58, 90], thus it could be accepted that also in the corneal epithelium these receptors could be represent the most obvious candidates for the chloride transport, especially the 5-HT_2_ subtype [56] playing their major role in the regulation of fluid transport or corneal homeostasis.

### 2.2. Iris and Ciliary Body

Serotonin is present in mammalian iris-ciliary body complex (ICB) at higher concentration that in non-mammalian species [5, 45, 73, 129, 137]. Moreover, the presence of serotonergic nerves has been demonstrated in studies conducted on the ICB of various species [102, 137, 138]. Experimental evidence and radioligand analyses have defined the presence at this level of three different types of serotonin receptors [10, 28, 85, 136, 137], i.e. 5-HT_1A_, 5-HT_2A/2C_ and 5-HT_7_ [98], one linked to a stimulation of inositol phosphates (5-HT_2_ subtype), while the others two are linked to cAMP activity.

The plausibility of the existence of more than one 5-HT receptor type in the ciliary body is confirmed by intraocular pressure (IOP) experiments. Topical application of serotonin has been reported to both elevate [84] and lower [88] IOP in rabbit. A large number of reports have shown that serotonin agonists and antagonists can produce increases and decreases in IOP when given orally, topically to the eye or when they are directly injected in the anterior chamber [10, 26, 34, 35, 64, 75, 84, 87, 88, 112, 113, 126, 136, 137]. A rationale for such apparently contradictory results may be due to the different sites of action, i.e. which class of serotonin receptor is activated. In fact, in rabbit the administration of 5-methyl-urapidil (a combined 5-HT_1A_ agonist/α_1_ adrenoreceptor antagonist) and 8-OH-DPAT (8-hydroxydypropylaminotetralin, a 5HT_1A_ agonist) reduces IOP, while the administration of 5-CT (5-carboxamidotryptamine, a 5-HT_1A_ and 5-HT_7_ agonist) increases IOP [36, 84, 99].

Chidlow, Le Corre and Osborne [28] have recently demonstrated that, in section taken through the whole eye and ciliary body, prominent 5-HT_1A_ and 5-HT_7_ receptor messenger ribonucleic acid signals were obtained. These signals were evident in both the pigmented and non-pigmented epithelial cell layers of the pars plicata region of the ciliary processes, but not in the pars plana or in the ciliary musculature. 5-HT_1A_ and 5-HT_7_ signals were apparent in the posterior processes but not in the iris processes. The presence of both receptors in the ciliary body would certainly provide an explanation for the shallow displacement curves observed in [^3^H]5-HT binding studies with the tissue [110], since this ligand can be used to label both receptors. Because the ciliary epithelium of the pars plicata is responsible for the secretion of aqueous humor, an obvious function for these two receptors would be an involvement in the control of aqueous production and, consequently, of the IOP level. Further, the almost identical distribution of the 5-HT_1A_ and 5-HT_7_ messengers ribonucleic acid indicate that the receptors may be co-localized in epithelial cells. The presence of two serotonin receptors with opposing effects on cAMP in the same cell layer prompts the suggestion that they could act antagonistically. The agonism of 5-HT_1A_ receptors, negatively coupled to cAMP, reduces IOP by decreasing the production of aqueous humor, like β-receptor antagonists which, diminishing the content of cAMP at the postjunctional site, lowers the aqueous humor secretion with a consequent decrease in IOP [98]. On the contrary, the administration of 5-CT induces a rise in IOP, which is partly or entirely caused by an increase in aqueous humor secretion mediated by 5-HT_7_ receptors [84].

The other type of serotonin receptor present in the ICB is a 5-HT_2_ type. Serotonin stimulates the accumulation of inositol phosphates in the ICB and this effect is partially counteracted by the 5-HT_2_ antagonists ketanserin, methysergide and mianserin [98]. Studies with ketanserin have demonstrated that, when orally or topically applied, it lowers IOP in animals, healthy volunteers and in glaucomatous patients [26, 34, 37, 64, 75, 88, 113, 126]. It has been emphasized that ketanserin also possesses an affinity for α_1_-adrenoreceptors [27, 36, 139], and for this reason the effects of ketanserin on IOP may not be entirely caused by its action on 5-HT_2_ receptors. However, data from human studies conducted after oral or topical administration of ketanserin, in which were determined the variations of IOP, total outflow facility and pupil diameter, demonstrated that the α_1_-adrenoreceptor blocking effect exerted by ketanserin should represent a further aspect of its mechanism of action, probably due to a functional sharing of these receptors [64, 87]. Lastly, already in 1992 Martin and co-workers showed the occurrence of a significant correlation between the content of serotonin in the aqueous humor and IOP in the human eye [85].

In 1981, Moro and his collaborators found that intravitreal injection of 5, 6-dihydroxytryptamine, a serotonergic neurotoxin, causes miosis [91]. The identification of 5-HT_7_, but not of 5-HT_1_ receptors in the rabbit iris, suggests that this population of serotonergic receptors is involved in the relaxation of the sphincter of the pupil. In fact, one of the function correlate to 5-HT_7_ receptor activation includes smooth muscle relaxation observed in a variety of isolated tissue preparations, in which elevation of cAMP concentration was also detected [3, 44]. Further evidence for the mediation of the relaxant response *via* the 5-HT_7_ receptor is provided by the localization of messenger ribonucleic acid transcripts encoding the 5-HT_7_ receptor in many blood vessels [67]. This hypothesized mechanism of action is also supported by the fact that various other receptor types, also positively coupled to cAMP, in the iris cause relaxation of the sphincter muscle [1, 28].

### 2.3. Lens

The presence of serotonin and its receptors in the lens has not been extensively investigated, even is it has been ascertained that 5-HT is both synthesized *in situ* and transported from the aqueous humor. Serotonin inhibits the ATPase at the level of lens epithelium, and the physiological activity of this membrane enzyme represents a crucial point in the maintenance of lens transparence [24]. More recently, the administration of a selective 5-HT_3_ antagonist to pregnant rats has been responsible for the development of nuclear cataract in the offspring. These findings seem to suggest that 5-HT_3_ receptors play a significant role during the lens organogenesis, throughout a triggering effect on the cation channels [66, 78]. Evidence for the presence of phosphoinositide cycle and its involvement in cellular signal transduction in the rabbit lens has been obtained by Vivekanandan and Lou [140]. These researches demonstrated that lens epithelium cells, like other cell types, possess a complete and functional phosphoinositide cycle, which is activated when the lens cells are exposed to serotonin or to other substances like calcium, epithelium growth factor and glucagon [140]. It is conceivable to suppose a role for the 5-HT_2_ receptor class in the regulation of this mechanism. Lastly, Chidlow and co-workers has found very low levels in the lens of messenger ribonucleic acid for 5-HT_1A_ and 5-HT_7_ receptors, indicating that these receptors are expressed in epithelium [28].

### 2.4. Retina

Although 5-HT is found in the mammalian retina only at low levels, considerable evidence suggests that it plays a role in visual processing. Pharmacological experiments indicate that numerous receptors for 5-HT are present in the mammalian retina [115, 116]. In the rabbit, where the most extensive study of mammalian retina has been performed, early radioligand binding experiments provided evidence for the presence of 5-HT_1_ and 5-HT_2_ receptor binding sites [89]. In addition, 5-HT_3_ binding sites have been characterized in the rabbit retina [6], and two subunits for this receptor (5-HT_3A_ and 5-HT_3B_) have been identified both in the nervous system of rabbit and rat, and in human retina. Further immunoreactivity studies have demonstrated that this receptor is localized specifically to the rod photoreceptor terminals of all three species [116]. Conflicting data existed about whether the 5-HT_1A_ receptor family was positively [9, 21, 28, 99] or negatively coupled to cAMP [43]. This question was solved by the cloning of the 5-HT_7_ receptor which shows a pharmacologic profile similar to that of the 5-HT_1A_ receptor, and is positively coupled to cAMP [81, 122]. These findings have allowed the identification of 5-HT_7_ receptor also in the retina [106, 117], confirming the suggestion that the retina serotonergic system may be a common feature in mammals despite the fact that the 5-HT levels are extremely low.

Neuro-pharmacological studies have shown that the activation of 5-HT_1A_ and 5-HT_2_ receptors affect visual processing [19, 20, 28], and 5-HT_2A_ receptors are associated with terminals of the photoreceptors and rod bipolar cells in the rabbit retina [117]. Recently, Osborne and co-workers have focused their attention on the possible role of serotonergic receptors of the retina in the modulation of the ganglion cells death, which plays a key role in the progression of the field loss in glaucomatous patients [110]. These authors have experimentally demonstrated that 5-HT_1A_ agonists are able to reduce IOP, through a 5-HT_1A_ receptors stimulation of the ciliary processes [29, 98], and to attenuate ganglion cell death induced by increased IOP, through interaction with membrane sodium channels and/or 5-HT_7_ receptors [104, 131, 134]. The clinical implications of these experimental evidence is yet to be demonstrated, but this studies seem to indicate that 5-HT_1A_ agonists could become a new class of drugs for the treatment of glaucoma in a near future [107].

The studies conducted by Nash, Osborne and co-workers have documented the presence of 5-HT_2A_ receptors at the level of the cultured rat retinal pigment epithelium cells. Moreover, human retinal pigment epithelium cells possess functional 5-HT_1A_ receptors which inhibit the cAMP production; even if the role of these receptors is unknown, the extensive range of metabolic actions mediated *in vitro* by cAMP indicates that they may modulate several cellular functions of the retinal pigment epithelium [94 - 96].

### 2.5. Vascular Structures

Before considering the interactions among serotonin, vascular compartments controlling the blood supply to the eye, and their hemodynamic characteristics, it is important to highlight that several structural differences exist between the extraocular vessels and the intraocular microvessels. Extraocular vasculature derives from the internal carotid artery including ophthalmic, lachrymal and ciliary arteries together with their correspondent draining venous structures. Some ramifications of these arteries, i.e. short and long posterior ciliary arteries, anterior ciliary arteries and central retinal artery, perforate the sclera or the optic nerve to supply the iris, ciliary body, retina, choroid, anterior and posterior regions of the optic nerve providing, respectively, several peculiar microvascular compartments: arterial circles of the iris, ciliary process, retinal vasculature, choroidal lobules, perioptic nerve arteriolar anastomoses (circle of Haller and Zinn) and pial microarterioles [100].

In experimental models, the role of 5-HT has been investigated considering its effects in some extraocular vessels, such as central retinal artery, ciliary artery and posterior ciliary arteries. These studies have documented a serotonin-induced vasoconstriction at the level of the posterior ocular segment [14, 57, 62]. In particular, Haefliger, Flammer and Luscher have demonstrated, in the porcine eye, this vasospasm was inhibited by ketanserin (an α_1_-adrenoceptor and 5-HT_2_ receptor antagonist) [57]. Hayreh and co-workers findings revealed that, although serotonin has no significant effect on ocular vessels of normal monkeys, in atherosclerotic ones it caused vasospasm of central retinal artery and/or posterior ciliary artery without inducing vasoconstriction of the retinal arterioles [60, 62]. More recently, in patients affected by normal pressure glaucoma, it has been identified a dysfunction of the systemic vascular endothelium in the course of its contractile responses to 5-HT and endothelin-1, suggesting alterations of the receptor populations on this endothelium [22].

## 3. PHYSIOPATHOLOGY OF THE INTRAOCULAR PRESSURE

**Fig. (4):**
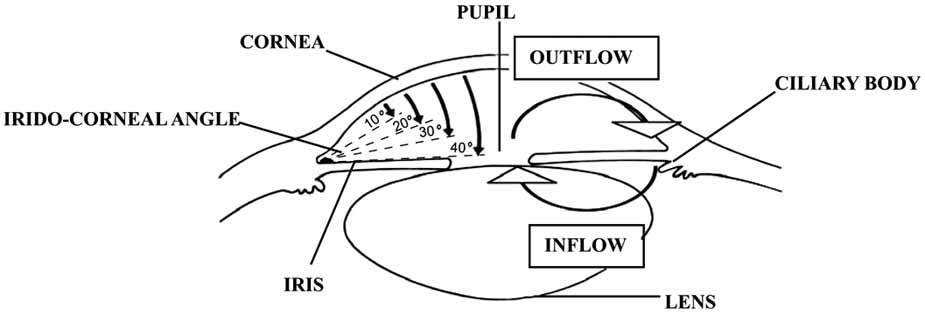
Schematic horizontal section of the anterior segment of the human eye.

Aqueous humor is a clear liquid contained into the anterior and posterior chambers of the eye (Figs. **3** and **4**). It is formed continuously, at a rate of approximately 2-3 μl/min, by the ciliary body in the posterior chamber [41]. Ciliary body is divided into two parts: posterior and anterior. The posterior part constitutes the flat ciliary ring and, because of its morphology, is also called pars plana ciliaris; the anterior one, which plays the active role in the secretion of aqueous humor, is made out of approximately 70 radial *plicae*, i.e. the ciliary processes. These are made of vascular bundles surrounded by a stroma of loose connective tissue and covered by two layers of ciliary epithelium. More in details, each ciliary process consists of a pigmented layer, which is continuous with the retinal pigment epithelium, and of a non-pigmented layer, which is continuous with the neuroretina [41, 92].

Aqueous humor composition is considerably different from the plasma, from which the aqueous humor derives. In fact, the concentration of chlorides, lactates, sodium, uric acid, are greater in the aqueous humor than in plasma, and above all ascorbate, whose concentration is almost 70 times greater than in plasma. Whereas, iron, glucose, cholesterol, creatinine, calcium, bilirubin, tryglycerides and, particularly, total proteins are lacking in the aqueous humor [41]. It is produced in two different ways: active and passive secretion. Approximately 80% of aqueous humor is secreted by the non pigmented ciliary epithelium through an active metabolic process involving a number of enzymatic systems, the most important of which is Na^+^/K^+^ ATPase pump, which secretes Na^+ ^ions into the posterior chamber. A probable linkage of this system with an active transport of anions, such as chloride and bicarbonate, is suggested by the prominent action of the carbonic anhydrase inhibitors on the rate of formation of aqueous humor, and also by the short-circuit studies of Holland and Gipson, which indicated that a part of the net flux of chloride could be accounted for by an active transport of this ion [8, 11, 55, 76]. The remaining 20% of aqueous is produced by passive processes, such as ultrafiltration and diffusion, which depend on the level of blood pressure in the ciliary capillaries, the plasma oncotic pressure and the level of IOP. With reference to this, it is important to remember, for an example, that when IOP increases, the rate of aqueous humor secretion diminishes [13]. Aqueous humor exerts several functions, the most important of which can be identified in the followings: (i) hydrostatic function, as it determines the ocular pressure which, in turn, optimizes optical functions; (ii) optical function, as the aqueous humor maintains both the cornea’s curvature and the refractive index of the corneal stroma; (iii) nutritional function for the cornea and the lens, as a carrier of nutrients and drawing off their metabolic end products; (iv) immunologic defense function, as a carrier of antibodies and drugs [127].

Aqueous humor flows from the posterior chamber through the pupil into the anterior chamber and then into the trabecular meshwork (Fig. **4**). The meshwork is essentially a filter that functions as a one-way valve. The ciliary muscle has insertions on the meshwork and ciliary contraction helps pump aqueous through the mesh, increasing the rate of drainage. In the irido-corneal angle of the anterior chamber, the aqueous enters the canal of Schlemm and then drains into episcleral veins. Small amounts of aqueous humor also leave the eye between the bundles of the ciliary muscle and through the sclera. Thus, aqueous humor leaves the eye by two routes: mainly, through the trabecular meshwork and Schlemm’s canal (conventional outflow), and secondary, through the anterior uvea (unconventional outflow) which, under normal circumstances, accounts for about 20% of the total outflow facility [8, 11, 41, 76, 92].

The level of IOP is determined by the balance between the rate of aqueous humor production (inflow) and the resistance that it encountered in the outflow pathways. If all these mechanisms working well, the fluid produced inside the eye equals the amount of fluid leaving the eye, and the resultant IOP is normal. Any alteration in the physiology of aqueous humor formation and drainage affects the IOP either increasing or diminishing its level. Thus, the factors able to affect IOP are: (i) rate of aqueous humor secretion; (ii) resistance encountered in outflow channels; (iii) pressure at the level of the episcleral venous. The relationship between these factors is expressed by the Goldmann equation as follows:

Po=FC+Pe

where P_o _= IOP (mmHg); F = rate of aqueous humor formation (μl/min); C = facility of aqueous humor outflow (μl/min/mmHg); P_e_ = episcleral venous pressure (mmHg) [52].

In the normal population mean IOP shows a Gaussian-like distribution curve skewed to the right. Mean IOP increases with age and is slightly higher in men (15.14 mmHg) than in women (14.94 mmHg) [16]. There is a wide range of IOP in normal population. Within this normal range, the upper of the IOP spectrum (20 mmHg) may be twice as high as the lower end of the spectrum (10 mmHg). Both values represent statistically normal pressures. Although there is not absolute cutoff point, 21 mmHg is generally considered as the upper limit of normal and values above this level are considered suspicious. In humans, has also been described a circadian slight, but significant, variation in IOP values, which are in general highest in the early morning and lowest during the night [41]. The amplitude of this fluctuation is small, ranging from 2 to 5 mmHg [114]. According to Ericson the phenomenon is due to a diurnal variation in the rate of secretion of fluid [47]. In fact, normal subjects have an increased probability of low IOP during the night and peaks at 4:00 a.m. and at the midnight, whereas between 8:00 a.m. and 8:00 p.m. the probability of occurrence of IOP changes is fairly low [72]. This means that an IOP value obtained from only one measurement throughout 24 hours is not sufficiently probative for a sure diagnosis. Thus, in passing from the physiology to the pathology of IOP, we are introducing certain concepts about glaucoma. This eponym is commonly used to define a wide range of diseases, which traditionally include conditions associated with increased IOP. In fact, classically, glaucoma is defined as "an eye disease ... characterized by increased IOP, excavation and degeneration of the optic disc, and typical nerve fiber bundle damage, producing defects in the field of vision" [15]. Actually, the glaucomatous disease is best defined as "... a related group of clinical syndromes characterized by damage of the optic nerve and loss of visual function obeying a characterized pattern usually associated with a statistically defined elevated IOP" [15]. Otherwise, it has been ascertained that in glaucomatous patients the reached IOP values exceed those individually tolerated by the optic nerve head; the inclusion of this important concept has resulted in a new definition of the disease: "glaucoma means a condition in which the optic nerve is damaged by IOP higher than the eye can tolerate" [15]. Although not all patients with glaucomatous optic neuropathy (GON) have an increased IOP and not all subjects with IOP elevation are affected by GON, the increased IOP remains the most important risk factor for its development [49]. The European Glaucoma Society has defined glaucoma as a etiologically heterogeneous group of ocular disorders characterized by the presence of an axon damage at the ganglion cells level, which lead to: (i) retinal nerve fiber layer alterations; (ii) optic nerve head cupping; (iii) typical visual field defect [48].

In the natural history of glaucoma, the progression of visual field loss is a direct consequence of GON. Two opposed pathogenetic hypotheses have been claimed to explain the peculiar glaucomatous findings (optic nerve head changes and visual field deterioration): a direct mechanical theory and an indirect vascular theory. In the first, the increased IOP directly damages the lamina cribrosa and neural axons. The second theory postulates that GON is the consequence of insufficient blood supply to the optic nerve either due to increased IOP or other pathologic conditions which impair the ocular microcirculation. The hemodynamic conditions, both systemic and local, affecting the microvascular structure, represent the second major risk factor, after increased IOP, involved in the progression of the GON and visual dysfunction [49, 144]. The blood supply to the eye derives primarily from the first branch of the internal carotid artery: the ophthalmic artery. In the orbit it branches into the central retinal artery, one to five posterior ciliary arteries and several anterior ciliary arteries. The main part of the choroid is supplied by the short posterior ciliary arteries, whereas anterior ciliary arteries supply part of the peripheral choroid and the anterior uvea [59, 94]. Blood supply to the optic nerve head is very unique. From the anterior to the posterior tract, it is possible to distinguish four regions: (i) the surface nerve fiber layer receives blood from retinal arterioles; (ii) the prelaminar region, between the previous one and the lamina cribrosa, is mainly supplied by the fine centripetal branches from peripapillary choroid; (iii) the lamina cribrosa region is entirely supplies by centripetal branches of the short posterior ciliary arteries; (iv) the retrolaminar region, immediately behind the lamina cribrosa, which receives both a centripetal (the pial vascular plexus, supplied by the Haller and Zinn circle which in turn, is formed by the short posterior ciliary arteries) and a centrifugal blood supply (deriving from branches of the central retinal artery). This description makes evident that the main source of blood supply to the optic nerve head is due to the posterior ciliary arteries circulation, which are obviously the only involved in the GON occurrence (Fig. **3**) [61].

A very simple classification of glaucoma, based on the fashion by which aqueous humor outflow is impaired, results in the distinction between open-angle or angle-closure glaucoma, yet divided into primary and secondary forms, according to the pathogenetic mechanisms responsible for their occurrence (Fig. **4**). If the impairment of aqueous humor outflow is due to the presence of other ocular diseases (vascular obstruction, inflammation, tumor) the glaucoma is called *secondary*. In the absence of such specific causes, the glaucoma is called primary, either of angle-closure or open-angle type. Primary open angle glaucoma (POAG) is defined *idiopathic* because its peculiar pathogenetic mechanisms are yet unknown. Angle-closure glaucoma refers to the type in which the peripheral iris obstructs aqueous humor outflow by closing the irido-corneal angle [130]. In angle-closure glaucoma, as well as in congenital and secondary glaucomas, the cause/effect relationship between increased IOP and GON is clearly comprehensible. In fact, in these forms, the mechanical theory exhaustively explains how increased IOP alone leads to GON development: the elevated pressure is responsible for a progressive compression and posterior displacement of the lamina cribrosa (a complex, reticulated elastic tissue which normally supports the optic nerve head and through which 1-2 million retinal ganglion cell axons leave the eye to form the optic nerve). The axoplasmatic transport is impeded, directly inducing cell death [118]. However, also in these forms, a synergism between mechanical and vascular effects may occur, because increased IOP, reducing blood supply to the optic nerve, simultaneously decreases the trophic factors availability, which also induce tissue ischaemia and cell death [49]. When IOP is only slightly elevated or even normal, like in most of the POAG patients or in normal tension glaucoma, it becomes more difficult to explain the GON occurrence only as dependent by IOP. A wide range of important epidemiological conditions able to make the optic nerve vulnerable, or more vulnerable, to a given IOP level have been identified. This susceptibility of the optic nerve head increases with age, with genetic make up (race and family history both are risk factors) and with certain diseases able to impair the blood flow, known to constitute the so-called vascular risk factors [49, 60].

Both high and low systemic blood pressure have been indicated as risk factors for GON development. A clear and significant relationship between glaucomatous damage and systemic hypotension has been documented, in particular with episodes of nocturnal arterial hypotension. Although these episodes are obviously expected in diseases characterized by systemic hypotension, they also occur in glaucomatous patients treated for systemic hypertension, and at a rate highest than that estimated [63, 70]. Therefore, as we have to search for IOP peaks, we have also to search for blood pressure dips. The immediate consequence of this event has been the need to introduce a new investigation in glaucomatous patients: the 24-hour monitoring of blood pressure, to know the real weight of this risk factor in the pathogenesis of GON [33, 38].

Atherosclerosis is recognized as the chief cause of death in the United States and Western Europe, being the major culprit for cardiovascular and cerebral accidents. It occurs very frequently, and begins in childhood [133]. Atherosclerosis commonly involves the large arteries, although the association with central retinal artery occlusion and anterior ischemic optic neuropathy (diseases involving small size arteries) has been well-documented. The lesion is characterized by endothelial injury, proliferation of smooth-muscle cells, deposition of lipid material (atheromata) under the intima, followed by connective tissue proliferation. On the altered vessel surface platelet accumulation easily occurs, and thrombus may form [49, 60]. The vascular endothelium plays an active role in vasomotor function of both macro- and micro-vasculatures, including the maintenance of vascular tone and regulation of blood flow [22, 40]. Vascular tone results from a balance between the concentration of vasodilator (e.g. nitric oxide) and vasoconstrictor (e.g. endothelin) compounds at the level of the endothelial cells. In atherosclerosis the endothelium dysfunction leads to the following schematic events: (i) defective dilatation of affected vessels; (ii) accentuated response to vasoactive products released by activated platelets, like serotonin; (iii) increased degradation of nitric oxide, which further impairs vasodilatation [60]. Thus, numerous factors may affect the blood perfusion through an organ, e.g. optic nerve head; among them, autonomic nervous system, circulating hormones and endothelial cells layer seem to play the most important role in this regulation. This very complex regulation might be easily altered by a lot of local or systemic conditions, which lead to an inadequate arterial constriction or dilation when needed. This phenomenon is termed *vascular dysregulation* or *vasospasm* [49].

The vascular dysregulation synergistically acts together with increased IOP, or with an IOP level higher than the eye can tolerate, reducing the ocular perfusion. On the other hand, as already demonstrated for nitric oxide, the some compounds responsible for vascular dysregulation might also affect IOP [40, 143].

## 4. EFFECT OF SSRIS ON THE INTRAOCULAR PRESSURE AND THEIR POSSIBLE ROLE IN THE DEVELOPMENT OF OPTIC NEUROPATHY

The presence of 5-HT receptors in the iris-ciliary body complex (ICB), together with the presence of serotonin and its metabolites (5-hydroxyindolacetic acid and 5-hydroxy-tryptophol) in aqueous humor, give evidence for a role of this amine in the regulation of aqueous humor dynamics. Experimental studies have ascertained that 5-HT_1A_, 5-HT_2A/2C_ and 5-HT_7_ receptors are located at the level of ICB [10, 28, 85, 98, 136, 137]. SSRIs increase the serotonin availability for these receptors, which react as though they were stimulated by agonists. Thus, the knowledge of each receptor function make possible to understand, with less difficulties, what happen when increased levels of endogenous serotonin reach the structures of anterior and posterior chambers of the eye (Fig. **3**).

To date, only the 5-HT_7_ receptors have been identified at the iris level. These serotonergic receptors, when stimulated by agonists, are responsible for the sphincter muscle relaxation [28]. This dynamic pupillary action is also confirmed by the observation that metergoline and methysergide, both potent 5-HT_7_ receptors antagonists, even cause passive mydriasis [3, 91]. In eyes with primary or secondary peculiar structural configuration of the irido-corneal angle (elevated hyperopia, steeply convex peripheral iris insertion, regularly straight iris with narrow angle, exfoliation syndrome, ciliary muscle hyperplasia, iris and ciliary body cysts, abnormality in shape and/or dimension of the lens, etc.), i.e. with a biometric predisposition, the further reduction in width of iridocorneal angle induced by mydriasis, may block the aqueous circulation, with the possible development of intermittent angle closure or acute angle-closure glaucoma (Fig. **4**) [120].

The 5-HT_1A_ receptors selective agonism reduces IOP; cyclic AMP concentration, responsible for a reduced rate of aqueous humor production, within the epithelial cells (pigmented and non-pigmented) diminishes [117]. The serotonergic effect on this receptor subtype is counterbalanced by a concomitant agonism exerted on the 5-HT_7_ receptors, which induces a cAMP elevation within the epithelial cells, with an increase of aqueous humor production [28]. The simultaneous stimulation of both 5-HT_1A_ and 5-HT_7_ receptors, with opposing effects on adenylate cyclase in the same cell layer, is not expected to modify IOP. On the contrary, the administration of 5-carboxamidotryptamine, a 5-HT_1A_/5-HT_7_ agonist, increases IOP [36, 84, 98], suggesting that, perhaps, its serotonergic action is lopsided towards the 5-HT_7_ receptors.

The 5-HT_2A/2C_ receptors are also expressed in the ICB. The agonism on these subtype receptors presumably increases the ciliary body blood flow, amplifying the metabolic activities of the ciliary processes, with an enhanced production of aqueous humor. Recently, Tian and co-workers have demonstrated that 1-(2, 5-dimethoxy-4-iodophenyl)-2-amino-propane, a 5-HT_2_ receptor agonist, grows both urine volume and urinary Na^+^ excretion, secondary to renal vasodilatation [135]. Although the repercussion of 5-HT_2_ agonism on IOP has not yet been investigated, this mechanism could represent one of the most plausible to explain another way by means SSRIs increases IOP [37, 87]. In fact, recently, it has been demonstrated that intravenous administration of fluoxetine significantly raises IOP in rabbits. This effect occurs *via* serotonin plasma levels augmentation, confirmed by elevated excretion of 5-hydroxyindolacetic acid in urine, and is strongly inhibited by topical administration of ketanserin, one of the most selective 5-HT_2A_ receptors antagonist [51, 64, 66, 87]. The answer of the ciliary body to a direct (*via* 5-HT_7 _receptors) or an indirect (*via* 5-HT_2A _receptors) stimulation activity consists in an increase of the aqueous humor inflow. When the outflow pathway are not able to drainage the aqueous humor overproduction IOP shall raise, according to the Goldmann equation [52]. On the other hand, IOP itself, triggering a negative feedback on the aqueous humor secretion, is able to exert an inflow autoregulation, which accounts for about 20% of aqueous humor production (passive ultrafiltration) [41]. The empirical correlation of this statement is in the evidence that fluoxetine administration to patients surely unaffected by glaucoma causes a transient 21.95% IOP increase from baseline, followed by a came back to the pre-treatment values in few hours [37]. This event has been observed in young adult depressed, otherwise healthy, subjects after the first exposure to SSRIs, but no data are actually available regarding the long-term effect of these compounds on IOP. In other words, it has not yet been established whether the chronic assumption of SSRIs is responsible for a iatrogenic IOP shift towards highest levels, with a consequent overload of the autoregulation system. This last mechanism can work by influencing only the F component of the Goldmann equation (rate of aqueous humor formation), whereas it is well known that the role played by the C component (facility of aqueous humor outflow), which decreases with aging, is also crucial for the IOP modulation. After SSRIs intake the ocular autoregulation system is able to ensure an equilibrium among the components affecting IOP homeostasis in the most part of depressed patients. This probably occurs just in the youngest subjects. On the contrary, in elderly patients the amount of plaque material at the level of the cribriform layer of the trabecular meshwork, derived from the sheaths of the elastic-like fibers, increases with age, inducing a parallel physiological increase in the outflow resistance. Moreover, in comparison to normal eyes, this material is significantly greater in cases of POAG and of intermittent narrow-angle glaucoma [83], suggesting that the enhanced resistance to the outflow observed in elderly patients and, at highest extent, in the subjects suffering from the two above mentioned forms of glaucoma, represents one of the mechanism leading to IOP increase. These mechanisms, at least theoretically, explain how the SSRIs intake can affect the IOP homeostasis and how the IOP ocular autoregulation system in elderly subjects, such as - independently from age - in glaucomatous patients or in eyes with primary or secondary peculiar structural configuration of the irido-corneal angle, could be not able to ensure the maintenance of IOP within individual acceptable physiological limits.

In 1991, Ahmad firstly described a 35-year-old man with acute angle-closure glaucoma occurred after five-week oral fluoxetine administration [4]. To date, other similar dramatic observations secondary to the intake of this specific SSRI have not been recorded. However, six cases of angle-closure glaucoma attack, induced by paroxetine administration, have been reported in the Literature [12, 18, 46, 71, 79, 80]. In the first three case reports, this adverse ocular reaction occurred in patients whose ages ranged from 70 to 91 years, an atypical life-period for the occurrence of acute-angle glaucoma, as confirmed by epidemiological studies [16]. In these elderly phakic patients, the lens status *per se*, in presence of an ocular biometric predisposition, synergistically interacting with paroxetine-induced passive mydriasis (*via* 5-HT_7_ receptors), easily can determine the glaucomatous attack [46, 71, 80]. The other three cases were observed in younger subjects with normal lens, but affected by hyperopia [12, 18, 79], an ascertained risk factor for intermittent angle closure or acute angle-closure glaucoma [120]. The interval between the onset of paroxetine administration and the development of the ocular adverse reaction was fairly different among these patients, ranging from few days to several weeks. There is only one case of acute attack of glaucoma reported in the Literature secondary to the use of fluvoxamine [69]. In this 66-year-old woman with a narrow-angle glaucoma, the exacerbation of the ocular disease became evident immediately after she started the antidepressive treatment. The two cases reported after citalopram administration have occurred in relatively young women, who were unaffected by neither cataract nor hyperopia [39, 86]. The mechanisms by which citalopram has been responsible of the acute attack has not been completely ascertained. Nevertheless, it may be possible that these patients were predisposed to the development of a glaucoma’s acute attack due to unknown inherent factors. About the causal relationship, it could be proposed that citalopram may directly act on the iris or ciliary body muscle through serotonergic or cholinergic mechanisms, or both. Lastly, a case of a 41-year-old woman who developed acute bilateral angle closure glaucoma with choroidal effusions while receiving escitalopram therapy has been recently described [145]. Medication-induced uveal effusion causing angle closure has been well documented with the antiepileptic medication topiramate [50], and in this case the mechanism appears to be similar. In fact, the ultrasonographic findings of choroidal effusion with ciliary body detachment during the acute attack with resolution of these findings once the attack was broken provide clear evidence that the mechanism of bilateral angle closure was related to uveal effusion. To date, no similar adverse events have been still documented after the administration of sertraline, although a significant increase in pupil diameter after administration has been documented with the use of these compounds. Mydriasis occurs quite rapidly following a single acute dose of sertraline, reaching a maximum 5 h after first administration. Subsequently, mydriasis remained stable at this level throughout the treatment period [123, 125]. In all these case reports, the Authors emphasized the role of passive mydriasis as precipitating event in the determinism of the acute angle-closure glaucoma [4, 12, 18, 39, 46, 69, 71, 79, 80, 86, 145]. The greater number of angle-closure glaucoma attacks reported with paroxetine, as compared to the other SSRIs, may be due to its relatively low IC_50_ value for the noradrenaline uptake (Table **3**). In fact, the sympathomimetic noradrenergic action, stimulating the dilator pupil muscle, produces an active mydriasis which, together with the serotonergic passive one, is able to reinforce the *crowding* at the level of the iridocorneal angle. This triggering mechanical phenomenon can bring iris tissue into closer proximity with the functional trabecular meshwork, the structure that normally allows aqueous humor to escape from the eye. For this reason, the use of TCAs, nonselective antidepressants, known to possess an evident noradrenergic action, is absolutely contraindicated in subjects with congenital and/or acquired predisposition to intermittent, sub-acute or acute angle-closure glaucoma [74, 81, 111, 120]. All data concerning the IOP modifications induced by SSRIs are summarized in Table **4**. 

The incidence of ocular side effects induced by SSRIs is probably underestimated because the IOP increase in the course of intermittent angle closure and, some more, in misdiagnosed forms of POAG is, respectively, pauci- or asymptomatic. Thus, it is not quite hazardous to hypothesize that the significant incidence of unspecified visual disturbances, reported by Preskorn in a comparative study on SSRIs tolerability [69], could be attributed - at least in some cases - to IOP modifications. This percentage probably comprises: (i) IOP impairments, related to intermittent angle closure or, more rarely, to episodes of marked IOP increase in patients with misdiagnosed POAG; (ii) acute reduction of retinal or optic nerve head blood supply, secondary to hemodynamic changes (vasoconstriction and/or platelet aggregation) at the level of central retinal artery or posterior ciliary arteries, respectively; (iii) locally vessel size dysregulation related to vasospasm of the ophthalmic artery in case of migraine with visual aura [37, 49, 60]. In clinical practice, during a antidepressive SSRIs treatment, the above mentioned modifications of the aqueous humor and/or blood dynamics are difficult to diagnose as much as to predict, because the real plasma concentration of both parent drug and its metabolites has a significant inter-individual variability. In fact, peculiar genetic mosaicism of cytochrome P450 (CYP) enzymes and/or several acquired pathophysiologic conditions, affecting the detoxifying processes, are able to delay the SSRIs biotransformation and elimination, prolonging their serotonergic effects in an unforeseeable fashion (individual peculiar SSRIs tolerability) [7, 23, 55, 141, 142].

Increased plasma levels of serotonin may be a factor or, more probably, a co-factor in the development of optic nerve head perfusional disorders, either synergistically with an IOP level higher than the eye can tolerate (individual critical IOP) or independently from IOP with a direct vascular dysfunction of the posterior ciliary arteries. In the course of chronic antidepressive treatment with SSRIs, multiple transient vasospasms at the level optic nerve head vessels may directly produce hemodynamic alterations that, time to time, could progressively induce a manifest optic ischemic neuropathy. In atherosclerotic individuals, the susceptibility to develop this kind of vascular disorder becomes highest because serotonin, enhancing platelet aggregation on the atheromata, can also indirectly trigger vasospasm of ocular arteries [60]. At the level of brain microcirculation it has been ascertained that serotonin is implicated in its regulation; significant changes in the status of 5-HT neurons of SNC have, in fact, vascular repercussion in specific brain regions. This implies that neuronal serotonin release constitutes the basis of normal blood flow regulation that, when dysfunctional, may lead to inadequate blood supply to the brain [31]. In coronary arterial district, the role of serotonin in vasoconstriction or even vasospasm has been recently indicated as possible co-factor in the occurrence of myocardial ischemia in patients with acute coronary syndromes [53]. In fact, some cases of vasospastic angina do not achieve response to the conventional treatment (Ca^2+^ antagonists, nitrates and K channel opener), whereas the association with serotonergic receptor blockade reduces anginal attack, suggesting that the vasospastic disorder is partially caused by serotonin [128]. The recently recorded escitalopram-induced uveal effusion [145] and sertraline presumed maculopathy [128] may be due to this blood flow dysfunction secondary to SSRIs administration.

Although AACG is an uncommon disease, risk factors have been identified. Women are more likely to develop AACG than men. Persons older than 50 years are at slightly increased risk, as are individuals with hyperopia. Those with a personal or family history of angle-closure glaucoma are at increased risk for the disease. Finally, people of Eskimo or Asian descent have higher rates of AACG [17]. The reported AACG attacks secondary to SSRIs administration have been more frequent in women than in men (8:3; 72%:28%; women:men) (Table **4**). There are some evidences supporting this datum: i) peripheral anterior chamber depth is less wide in women than in men and, with aging, it tends to be progressively reduced; ii) the different prevalence of depression between the two sexes, with a 2:1 female: male ratio in adulthood [68]. Moreover, independently by sex, hyperopic eyes have a predisposition to a reduced wide of the angle, with a consequent enhanced risk for glaucoma acute attack [17, 32]. 

Lastly, SSRIs intake has been associated with the syndrome of inappropriate antidiuretic hormone secretion (SIADH) and hyponatremia [77]. The frequency of hyponatremia is around 8 per 1, 000 among elderly women. The risk of developing hyponatraemia, a potentially fatal condition that is typically asymptomatic until it becomes severe, appears to be highest during the first few weeks of treatment. SIADH is more likely in some populations, including people who are elderly or who take diuretics [120]. A dose-dependent elevation of IOP has been reported in rabbits after injections of vasopressin into the 3(rd) ventricle. This IOP elevation was blocked by the pretreatment with the V(1) receptor antagonist. Thus, a third mechanism by which SSRIs might affect IOP could be dependent by the central effect of ADH on IOP *via* stimulations of the V(1) receptors, without affecting pupil diameter [54].

## CONCLUSION

In the clinical practice, the currently available SSRIs represent the most widely utilized medical therapy in patients affected by depression and/or other heterogeneous mood and anxiety disorders. A worldwide prospective analysis revealed that, during the next two decades, the incidence of these psychiatric diseases in the general population is destined to progressively grow [93]; thus, unavoidably, the agreeable effectiveness-tolerability-safety profile of the SSRIs will result in a more extensive prescription of these antidepressants by psychiatrists and also non-psychiatrists. Until the real involvement of SSRIs on IOP increase and optic nerve head changes shall not be well-ascertained, it is difficult to draw final conclusions on the more reasonable clinical management of depressed patient under SSRIs treatment. The knowledge of individual tolerability, angle-closure predisposition and critical IOP could be goals no so difficult to realize. In fact, although in the course of a search utilizing Medline, we have found only eleven papers documenting cases of acute angle-closure glaucoma related to SSRIs administration [4, 12, 18, 39, 46, 69, 71, 79, 80, 86, 145], the World Health Organization Adverse Drug Reactions database contains several other reports of undefined glaucoma associated with the different marketed SSRIs. This discrepancy further confirms the suspicious that the recording of pauci- or asymptomatic side effects, like POAG, is underestimated. Thus, because experimental and clinical findings indicate that SSRIs affect pupil diameter, aqueous humor dynamics and optic nerve blood flow, the following recommendations should be taken into account:

- in presence of ascertained glaucomatous risk factors (sex, race, positive familial history for glaucoma, mid or high hyperopia, cataract, etc.) is ethic to submit the patient to an ophthalmologic consultation, before the starting of the SSRIs treatment;
- in elderly patients, before the starting and during the SSRIs treatment, serum electrolytes, and periodical IOP measurements should be prescribed; in presence of hemorheologic risk factors, specific examinations (i.e. computerized automated perimetry performed with different peculiar strategies and scanning laser Doppler flowmetry of the optic nerve head) should be, at least, advised.

## Figures and Tables

**Table 1:** Nomenclature Used to Identify Various Populations and Subpopulations of Serotonin Receptors (5-HT Receptor Sub-types) [2, 5, 7]

Receptor	Structural Family	Signal Transduction	Localization	Function	Comments
5-HT_1A_	GPCR	Inhibition of AC	Hippocampus, Septum, Amygdala, Cortical limbic areas, Raphe nuclei	Autoreceptor	Cloned and pharmacological 5-HT_1A_ sites.
5-HT_1B_	GPCR	Inhibition of AC	Basal and Trigeminal ganglia, Striatum, Frontal cortex Cerebral arteries and other vascular tissues	Autoreceptor	Rodent homologue of human 5-HT_1Dβ_ receptors.
5-HT_1D_	GPCR	Inhibition of AC	Basal and Trigeminal ganglia	Unknown	Newer name: 5-HT_1Bβ_; a mouse homologue of human 5-HT_1Dβ_ receptors; sites identified in binding studies using brains homogenates; 5-HT_1Dα_ and 5-HT_1Dβ_ (cloned human 5-HT_1D_ subpopulations).
			Cranial blood vessels	Vasoconstriction	
5-ht_1E_	GPCR	Inhibition of AC	Cerebral cortex, Striatum	Unknown	Newer name: 5-ht_1Eβ_; sites identified in binding studies using brain homogenates; 5-ht_1Ea_ (an alternate name for cloned human 5-ht_1E_ receptors).
5-ht_1F_	GPCR	Inhibition of AC	Dorsal raphe, Hippocampus, Striatum, Cerebral cortex, Thalamus, Hypothalamus	Unknown	A cloned human 5-HT_1_-like receptor population.
			Uterus, Mesentery	Vasoconstriction	
5-HT_2A_ (D receptor)	GPCR	Activation of PLC	Cerebral cortex, Claustrum, Basal ganglia	Neuronal excitation	Newer name: 5-HT_2A_; original "5-HT_2_" (5-HT_2α_) receptors
			Smooth muscle	Contraction	
			Platelets	Platelet aggregation	
5-HT_2B_	GPCR	Activation of PLC (Other unknown)	Cerebellum, Lateral septum, Hypothalamus, Medial amygdala	Unknown	Newer name: 5-HT_2B_; 5-HT_2_-like receptors in rat fundus
			Stomach fundus	Contraction	
			Blood vessels	Vasodilatation	
5-HT_2C_	GPCR	Activation of PLC	Choroid plexus	Vasodilatation	Newer name: 5-HT_2C_; original "5-HT_1C_" (5-HT_2β_) receptors.
5-HT_3_ (M receptor)	5-HT-GIC	Ligand-gated ion channel	Area postrema, Enthorinal and Frontal cortex, Hippocampus, Solitary Tract, Amygdala Peripheral pre- and post-ganglionic autonomic neurons, Sensory nervous system, Gastrointestinal tract	Neuronal excitation	An ion channel receptor.
5-HT_4_	GPCR	Activation of AC	Hippocampus, Colliculi, Nucleus accumbens Gastrointestinal tract, Vascular smooth muscle	Neuronal excitation	5-HT_4_ population originally described in functional studies; 5-HT_4s_ (short form of cloned rat 5-HT_4_ receptors); 5-HT_4L_ (long form of cloned rat 5-HT_4_ receptors).
5-ht_5A_	GPCR	Inhibition of AC	Hypothalamus, Hippocampus, Corpus callosum, Fimbria, Cerebral ventricles, Glia	Unknown	Cloned mouse, rat and human 5-ht_5_ or 5-ht_5A_-like receptors.
5-ht_5B_	Unknown	Unknown			
5-ht_6_	GPCR	Activation of AC	Caudate nucleus, Striatum, Amygdala, Nucleus accumbens, Hippocampus, Cerebral cortex and Olfactory tubercle	Unknown	Cloned rat and human 5-HT receptors.
5-HT_7_	GPCR	Activation of AC	Cerebral cortex, Thalamic nuclei, Sensory nuclei, Substantia nigra, Hypothalamus, Raphe nuclei Vascular tissues, Smooth muscle (ileum)	Unknown	Cloned rat, mouse, guinea pig and human 5-HT receptors; original "5-HT_1-_like" receptors.

**Legend:** GPCR, G protein-coupled Receptor; 5-HT-GIC, 5-HT-gated ion channel; AC, adenylate cyclase; PLC, phospholipase C.

**Table 2:** Distribution of 5-HT Receptor Subtypes in the Ocular Tissues and in the Vascular Structures of the Eye

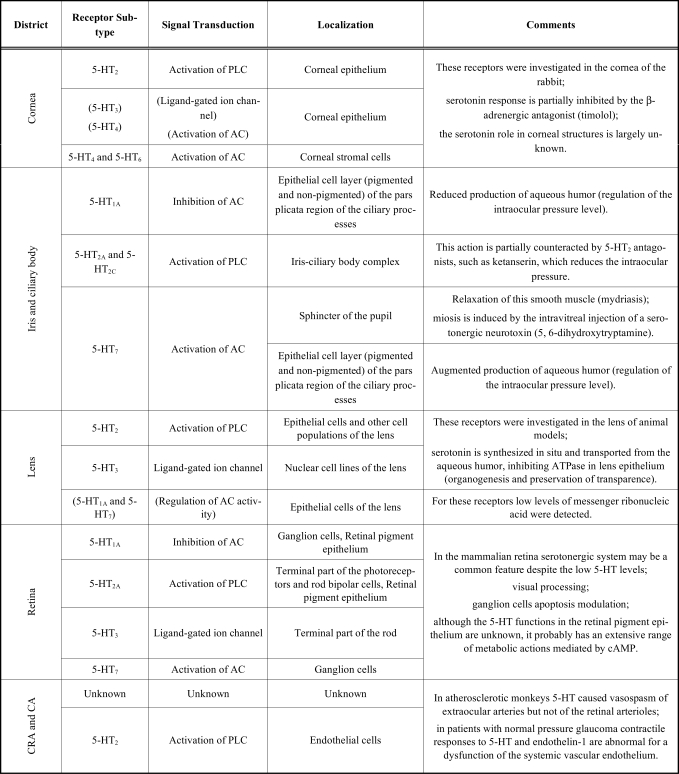

**Legend:** AC, adenylate cyclase; PLC, phospholipase C; cAMP, adenylate cyclase; CRA, central retinal artery; CA, ciliary artery.

**Table 3:** Pharmacologic Properties of the SSRIs: Their Effect on the Uptake of Biogenic Amines *in Vitro* (Adapted from: Hyttel J, 1994; Burke WJ & Kratochvil CJ, 2002) [160, 167]

SSRI	Monoamine Uptake Inhibition, IC_50_ value (nM)			IC_50_ NA Uptake / IC_50_ 5-HT Uptake
	Serotonin	Noradrenaline	Dopamine	
Fluoxetine	6.8	370	5000	54
Sertraline	0.19	160	48	840
Paroxetine	0.29	81	5100	280
Fluvoxamine	3.8	620	42000	160
Citalopram	1.8	6100	40000	3400
Escitalopram	1.5	2500	65000	1700

**Table 4:** Summary of the Reports Describing SSRIs-Induced IOP Modifications

Author	SSRI Administered	Patient’s Age and Sex	Side Effect	Interval from the Administration
Ahmad S. [4]	Fluoxetine	35-year-old man	Acute angle closure glaucoma	Five weeks
Costagliola C, *et al.* [37]	Fluoxetine	male: female 5:15 age range 33 – 47 years	IOP increase in both eyes	Two hours
Kirwan JF, *et al.* [71]	Paroxetine	91-year-old woman	Bilateral acute angle closure glaucoma	One day
Lewis CF, *et al.* [80]	Paroxetine	70-year-old man	Acute angle closure glaucoma	Three days
Eke T, Bates AK. [46]	Paroxetine	84-year-old woman	Acute angle closure glaucoma	Two weeks
Bennett HG, Wyllie AM. [12]	Paroxetine	53-year-old woman	Acute angle closure glaucoma	Three days
Browning AC, *et al.* [18]	Paroxetine	40-year-old man	Acute angle closure glaucoma	Two weeks
Levy J, *et al.* [79]	Paroxetine	54-year-old woman	Late bilateral angle closureglaucoma	Two months
Jimenez-Jimenez FJ,*et al.* [69]	Fluvoxamine	66-year-old woman	Aggravation of narrow-angle glaucoma	One day
Croos R, *et al.* [39]	Citalopram	54-year-old woman	RE: ocular hypertension LE: acute angle closure glaucoma	Soon after Citalopram and alcohol overdose
Massaoutis P, *et al.* [86]	Citalopram	55-year-old woman	Bilateral acute angle closure glaucoma	Three months
Zelefsky JR, *et al.* [145]	Escitalopram	41-year-old woman	Bilateral angle closure glaucoma.	Four weeks
